# Erythrocytes as Carriers for Drugs and Contrast Agents

**DOI:** 10.5195/cajgh.2013.89

**Published:** 2014-01-24

**Authors:** Mauro Magnani

**Affiliations:** Department of Biomolecular Sciences, University of Urbino, Urbino, Italy

**Keywords:** erythrocytes, biomedical engineering, red blood cell technology

## Abstract

Erythrocytes, also known as Red Blood Cells (RBC), are typically used in transfusion medicine to replace lost blood in patients who underwent different kinds of medical treatments as well as those involved in accidents resulting in blood loss. In addition to these common uses, RBC are being used for a variety of new applications either as therapeutics or as diagnostics. Most of these novel approaches are made possible due to the peculiar properties of these cells. We have invented a technology that allows cells to be opened and resealed without affecting their main physiological characteristics with a minimal amount of patient blood. Uses of processed RBCs in biomedical engineering include work with drugs, biomedical compounds and/or nanomaterials. These constructs are a new armamentarium available to the physicians for the release of drugs in circulation, for targeting drugs to selected sites in the body, or for in vivo diagnostic procedures based on magnetic and/or optical methods. Autologous human RBC loaded with dexamethasone (EryDex), a common corticosteroid, have been used in the treatment of Cystic Fibrosis, Crohn’s Disease, and other severe inflammatory conditions. Benefits and safety of this technology have been documented in over 2,500 treatments. EryDel SpA is a company focused on developing and commercializing innovative therapies and diagnostics based on the use of autologous RBCs as agent carriers. More recently, EryDel SpA completed a Phase II Proof of Concept study in patients with Ataxia Telangiectasia (AT), a rare progressive neurological autosomal recessive disorder that leads to mortality in most patients at an early age, with significant benefit seen on primary and secondary end-points. EryDex treatment has received Orphan Drug Designation by EMA for the treatment of Cystic Fibrosis and both by EMA and FDA for the treatment of AT.

The encapsulation of superparamagnetic nanoparticles within RBC has lead to the generation of new biomimetic constructs that now permits the use of these nanomaterials in vivo avoiding their rapid sequestration and their accumulation in unwanted districts (PCT WO 2008/003524 A3). Similarly, the encapsulation of infrared fluorescent agents into RBC gives opportunity to the measurement of vasomotion in the human retinal vasculature suggesting a possible correlation with retinal edema.

In summary, the newly developed RBC-based drug delivery system is an innovative technology platform that could be used in a wide range of applications opening to unlimited new therapeutic approaches. Furthermore, the same system has been adapted to deliver contrasting agents within the body enabling the improvement of current fluorangiographic procedures and the imaging by Magnetic Resonance (MRI) and Magnetic Particle (MPI). EryDel S.p.A. has recently completed trials to bring EryDex treatment to the market and to implement the clinical applications of the RBC technology.

